# A comprehensive image dataset for accurate diagnosis of betel leaf diseases using artificial intelligence in plant pathology

**DOI:** 10.1016/j.dib.2025.111564

**Published:** 2025-04-22

**Authors:** Rashidul Hasan Hridoy, Md. Tarek Habib, Imran Mahmud, Aminul Haque, Md Abdulla Al Mamun

**Affiliations:** a Department of Software Engineering, Daffodil International University, Dhaka, Bangladesh; b Department of Computer Science and Engineering, Independent University, Bangladesh, Dhaka, Bangladesh; c Department of Computer Science and Engineering, Daffodil International University, Dhaka, Bangladesh; d Department of Computer Science and Engineering, Hajee Mohammad Danesh Science and Technology University, Dinajpur, Bangladesh

**Keywords:** Betel plant, Agricultural dataset, Leaf disease, Crop disease, Disease classification, Computer vision, Deep learning

## Abstract

In South Asian countries, agriculture is a crucial employment field, and a remarkable number of people depend on it for their livelihood. Crop diseases are a significant threat to sustainable development in the agriculture field. Automated efficient crop disease diagnosis techniques developed with comprehensive field image datasets can play a vital role in preventing diseases at an early stage. Betel leaf is widely consumed in South Asian countries for its nutritional benefits, but to the best of our knowledge, no extensive dataset of betel leaf is available that can play a crucial role in developing accurate disease diagnosis tools. Farmers face a significant economic loss due to betel leaf diseases, and due to the lack of efficient diagnosis tools, the farming of betel leaf has become very difficult day by day. Our motive is to develop a reliable and versatile image dataset of field images that will assist artificial intelligence-based pathology research on betel leaf diseases. This dataset contains healthy leaf images and two common disease images of betel leaf such as leaf rot and leaf spot [1]. Initially, 2,037 betel leaf images were captured in a natural daylight environment from several betel cultivation fields in Bangladesh. Afterward, 10,185 images were generated using image augmentation strategies including flipping, brightness factor, contrast factor, and rotation. This dataset is well-compatible with machine learning and deep learning-based pathology research, as it contains enough image samples for model training, validation, and testing. Moreover, a comparison study is conducted that ensures this dataset fulfills the gap of a reliable and extensive dataset of betel leaf. This comprehensive dataset serves as a crucial resource for researchers in developing efficient computational models for accurate betel leaf disease diagnosis.

Specifications Table

**Table:** 

Subject	Computer Sciences
Specific subject area	Plant Pathology, Crop Disease Diagnosis, Leaf Disease Recognition, Image Classification, Computer Vision, Machine Learning, Deep Learning
Type of data	Images (.jpg)
Data collection	The 2,037 original images of this dataset were collected from four different betel cultivation fields from January 2020 to January 2022 manually using the camera of the HUAWEI Y7 Pro smartphone under the supervision of plant pathology experts. Images were collected at different seasons and categorized into three classes: healthy leaf, leaf rot, and leaf spot.
Data source location	Images were captured from the following places of the Mymensingh division in Bangladesh: 1. Charshihari, Ishwarganj, Mymensingh, Bangladesh. 2. Chorhossianpur, Ishwarganj, Mymensingh, Bangladesh. 3. Uchakhila, Ishwarganj, Mymensingh, Bangladesh. 4. Lakshmiganj, Ishwarganj, Mymensingh, Bangladesh.
Data accessibility	Repository name: Mendeley Data Data identification number: 10.17632/vpzkntzjty.1 Direct URL to data: https://data.mendeley.com/datasets/vpzkntzjty/1 The dataset can be accessed directly using the mentioned URL, a zip file of 1.28 GB will be downloaded. Researchers or interested individuals can use the images of the dataset by extracting the zip file.
Related research article	None

## 1 Value of the Data

• This unique dataset of betel leaf images is crucial for the crop-based economy of most South Asian countries, including Bangladesh. Due to the use of traditional approaches for disease diagnosis and management, farmers face a remarkable economic loss every year. This dataset will assist researchers in developing efficient leaf disease diagnosis tools for betel leaf.
• At the limit of our knowledge, there is a lack of a robust and comprehensive dataset of betel leaf images available for computer vision-based research and image processing. This dataset fulfills this gap, which creates the opportunity to conduct advanced research on disease diagnosis for developing automated disease management tools using machine learning (ML) and deep learning (DL) techniques.
• The images of this dataset are well structured into eight sub-folders and images were captured from fields under the supervision of plant pathology experts, which ensures its robustness and acceptance broadly. Moreover, the size of all images is 1024 × 1024 pixels which supports almost all types of computer vision-based techniques for image classification and analysis.
• This dataset contains 12,222 images of betel leaf, making it a good avenue for both the development and evaluation of artificial intelligence (AI) models for precision agriculture. Deploying these AI models with smartphone applications and IoT devices can change the current miserable scenario of betel leaf cultivation by recognizing diseases at an early stage. Diagnosing diseases at an early stage can prevent economic loss and will reduce the use of fertilizer, which is crucial for saving nature from fertilizer and pesticide pollution.
• The outcome of the comparison study ensures the novelty of this dataset strongly, as it overcomes the limitations of existing datasets. This betel leaf dataset can be used for educational purposes also, learners can gather deeper knowledge on computer aided-plant pathology. It also advances the alliance of computer vision and plant pathology that assists in achieving sustainability in the agricultural sector. This dataset is freely accessible worldwide and inspires researchers to develop efficient innovative systems for advancing agriculture.

## 2 Background

**Fig. 1:**
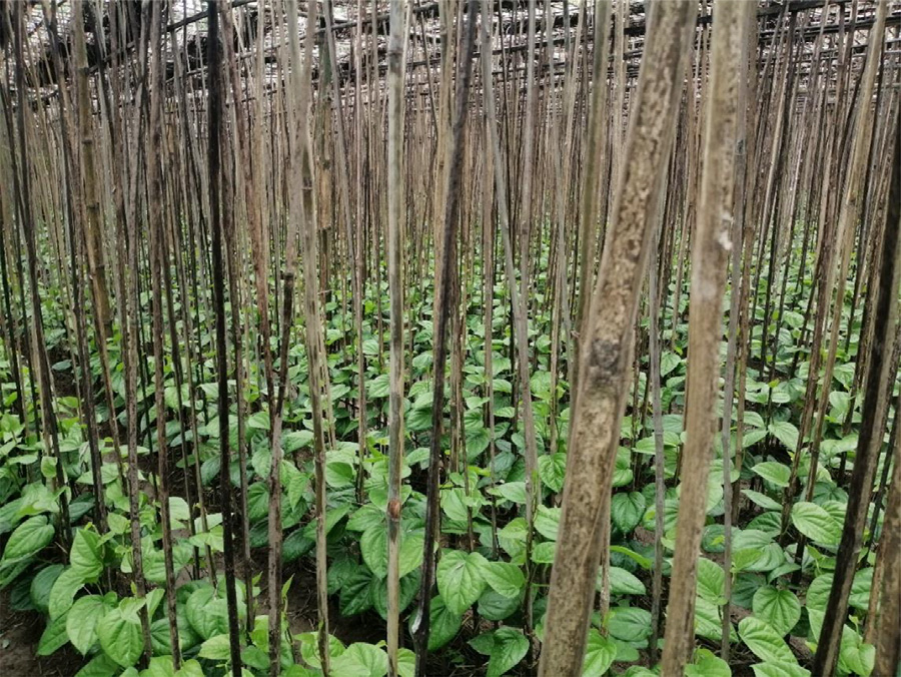
The betel field from where the original images were taken.

Betel leaf (*Piper betel L.*) is a traditional crop in Southeast Asia, its shiny heart-shaped leaf has several traditional uses and is also immensely renowned for its nutrients in the food and medicine industry [2,3]. This leaf is widely cultivated in South Asian countries and has a significant cultural value, and a remarkable number of people have been completely dependent on its cultivation for their livelihood since ancient times [4,5]. However, its cultivation is a troublesome and challenging task as this plant is highly prone to diseases and lacks proper disease management systems, causing financial loss and increasing the use of fertilizer and pesticides. ML and DL-based approaches are now widely used in the agricultural sector for crop disease management where leaf images are the primary building block for training, validating, and testing ML and DL models [6,7]. Pushpa et al. presented a dataset of several species of Indian medicinal plants, including 114 images of betel leaf [8]. Mohammad et al. addressed a dataset on betel leaf that contains 1000 original images [9]. Soumick et al. presented a dataset that contains 500 good and 500 bad images of betel leaf [10]. Milind et al. addressed a dataset that includes 4156 images of unhealthy betel leaves, and no images of healthy leaves provided [11]. The lack of an extensive and reproducible betel leaf dataset is a great obstacle in developing efficient disease diagnosis and crop management tools. This motivated us to create a comprehensive image dataset for AI-based pathology research on betel leaf. The betel leaf dataset contains 2,037 original images which are gathered from cultivation fields, and images were categorized with the help of pathology experts into three classes. Images were captured from four different locations in Bangladesh in different seasons under real environmental situations and several growth stages were considered, which ensures images contain disease symptoms properly. The 2,037 original images in the dataset ensure the reproducibility and transparency of the dataset, lay the crucial ground step for future development in betel leaf disease management, and contribute towards the production enhancement and sustainability of the agricultural field. Fig. 1 delivers the image of the betel cultivation field (Location: Charshihari, Ishwarganj, Mymensingh, Bangladesh).

## 3 Data Description

The betel leaf image dataset contains a total of 12,222 joint photographic experts group (JPEG) images, with dimensions of 1024 × 1024 pixels, both horizontal and vertical resolutions of 96 dots per inch (DPI), and a bit depth of 24. These 96 DPI JPEG images are highly suitable for the training of DL models that ensure models generalize perfectly and are also appropriate for computer vision, medical, and industry applications for their quick processing ability and efficiency. To ensure the clarity of images, images were collected during the daytime from 9 AM to 5 PM under different levels of natural lighting. To ensure diversity in the dataset, different climatic factors and the growth stage of plants were also considered during sample collection. Five different image augmentation techniques were utilized to generate 10,185 images from the original images captured from the cultivation field, and the file names of the images are in structured format, using this researcher or interested individuals can easily get basic information of the images. It is a multiclass image dataset, and Table 1 gives a brief description of each class with representative images.

**Table 1:** Description of the sample image of each class of the dataset.

Class Name	Description	Sample image
Healthy Leaf	A healthy leaf of betel is a vibrant green leaf with a glossy surface, which is unblemished and smooth. It contains visible venation; its texture is smooth and looks slightly waxy texture. This type of leaf doesn't contain any type of spot, and has no manifestation of discoloration, pest damage, or fungal infections [2,12].	
Leaf Rot	At the early stage, tiny water-soaked spots are seen in the heart-shaped leaf surface, the area of these spots increases day by day and turns into black or brown colors which form large patches. Moreover, disease-affected areas of the leaf surface become pulpy and decay quickly. Additionally, gray or white fungal areas are seen on the leaf surface under humid conditions [13].	
Leaf Spot	Small-scale round to uneven black or brown dots appears on the leaf surface in the primary stage of the disease. These dots are wet in the beginning and become dry slowly. Day by day, these dots cover a large portion of the leaf and expand. The existence of dark dots assures the infection of leaf spots. Moreover, repeated rainfall and severe humidity increase the expansion of black dots [14].	

**Fig. 2:**
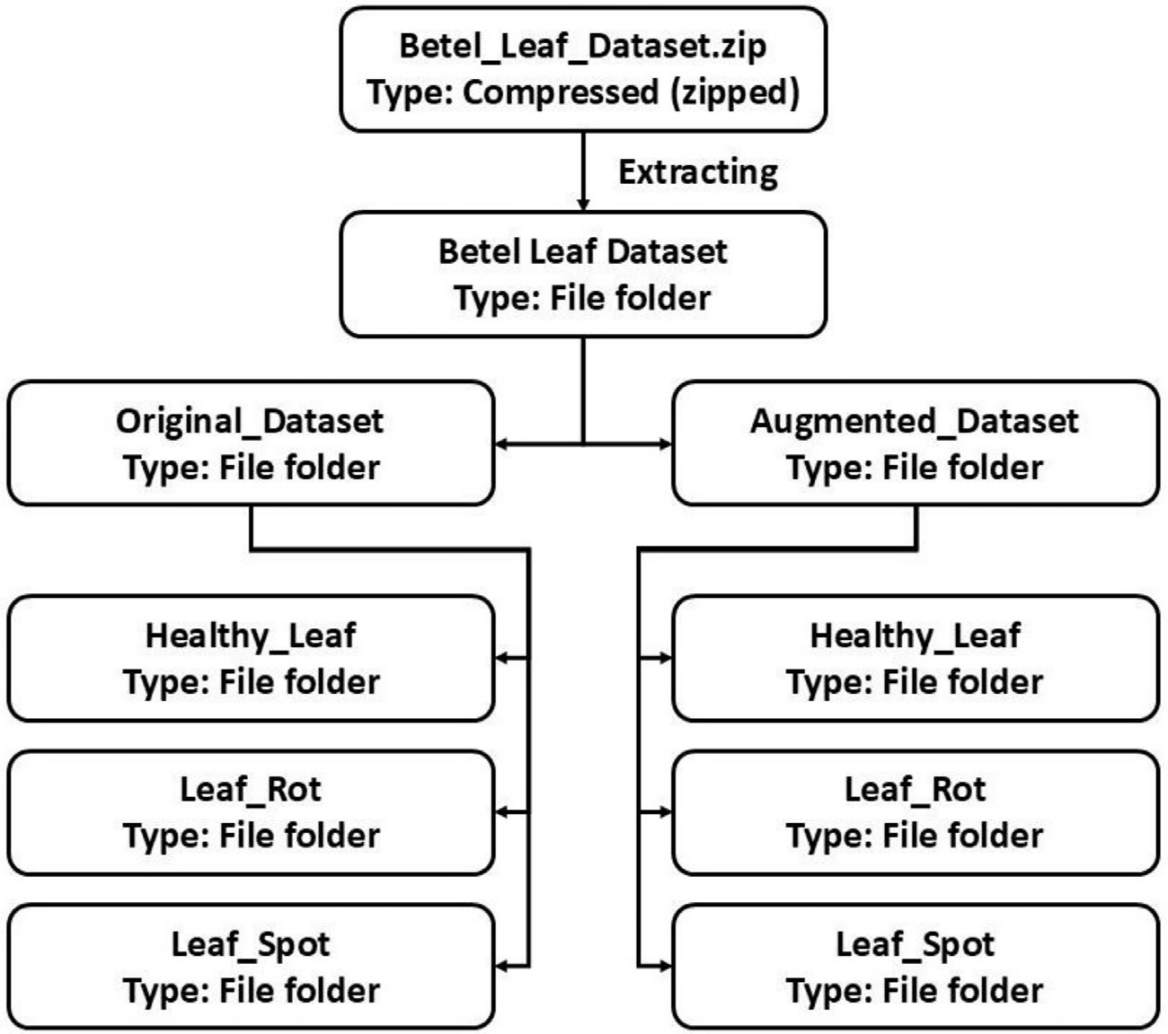
Organization of betel leaf disease dataset.

This dataset contains eight folders and 12,222 files whose size is 1.30 GB, and has two parts, one stores original images, and the other stores augmented images. Each of these parts contains three folders, where images of three classes are stored. The formation of the dataset directory is illustrated in Fig. 2. The image distribution of each class is as follows: Healthy Leaf: 53.02% (original), 53.01% (augmented), Leaf Rot: 13.20% (original), 13.20% (augmented), and Leaf Spot: 33.78% (original), 33.79% (augmented). Details of the original and augmented images are given in Table 2, Table 3 with folder names, respectively. Moreover, an in-depth comparison study is also carried out, in which the presented dataset is compared with the existing dataset of betel life, which is given in Table 4.

**Table 2:** Statistics of the original images.

Class name	Folder name	Number of original images
Healthy Leaf	Healthy_Leaf	1,080
Leaf Rot	Leaf_Rot	269
Leaf Spot	Leaf_Spot	688

**Table 3:** Statistics of the augmented images.

Class name	Folder name	Number of augmented images
Healthy Leaf	Healthy_Leaf	5,400
Leaf Rot	Leaf_Rot	1,345
Leaf Spot	Leaf_Spot	3,440

**Table 4:** Comparison with existing datasets of betel leaf.

Dataset	Number of original images	Number of augmented images	Remarks
Pushpa et al. [8]	114	Not mentioned	This dataset contains only 114 images of betel leaves, and no disease-affected image is presented which has made it unsuitable for disease diagnosis.
Mohammad et al. [9]	1000	2589	This dataset has less variety of symptoms of the disease as it contains fewer original images of betel leaf which decreases model generalization capability.
Soumick et al. [10]	1000	Not mentioned	This dataset contains good and bad images of betel leaf, no disease-affected images are present here, which makes this dataset unsuitable for disease diagnosis.
Milind et al. [11]	4156	Not mentioned	This dataset contains only disease-affected images of betel leaf, no healthy images are present here, which makes this dataset unsuitable for disease diagnosis.
This dataset [1]	2,037	10,185	This dataset provides more original images than other mentioned datasets which ensures enough diversity of disease symptoms presented in the dataset that increases model generalization ability. Also, more real images make the dataset reproducible, and the number of augmented images is higher than other datasets.

## 4 Experimental Design, Materials and Methods

**Fig. 3:**
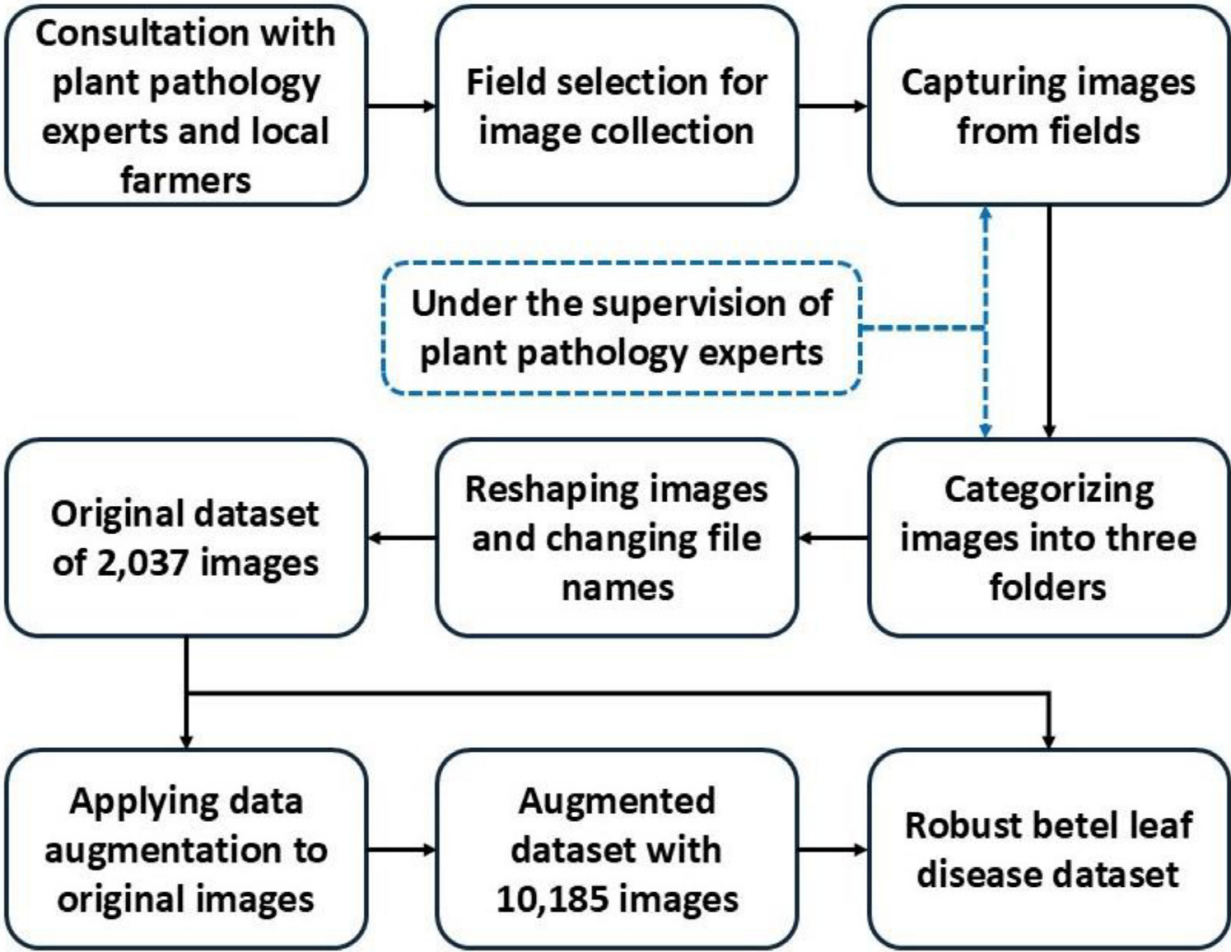
The steps followed during the dataset development.

Properly collecting images is mandatory for developing robust datasets for leaf image diagnosis applications. Quality datasets are key to efficient ML and DL applications in computer vision, and a powerful correlation exists between the performance of computer vision applications and robust datasets. To ensure robustness and quality, several key steps were followed during the dataset development process which are illustrated in Fig. 3. In the beginning, consultation with plant pathology experts and local farmers was carried out. Expert pathologists provide instructions to identify images based on symptoms, and farmers provide their traditional knowledge and experience with betel leaf diseases. With the help of local farmers, four betel cultivation fields were chosen for capturing images, and experts also visited fields to monitor the collection process. The image collection duration was 24 months, and captured images were categorized into three classes under the supervision of pathologists. All images were captured with the default camera setup and no additional brightness and contrast were used; the details of the used camera are provided in Table 5.

**Table 5:** Specifications and configuration details of the camera used.

Title	Description
Manufacture	Huawei
Model	JKM-LX2
Main camera	13 megapixels
Depth-sensing camera	2 megapixels
Focal length	3.62 millimeters
F-Stop	f/1.8
Exposure time	1/100 sec
ISO	80
Exposure Bias	0
Flash Status	No flash

**Fig. 4:**
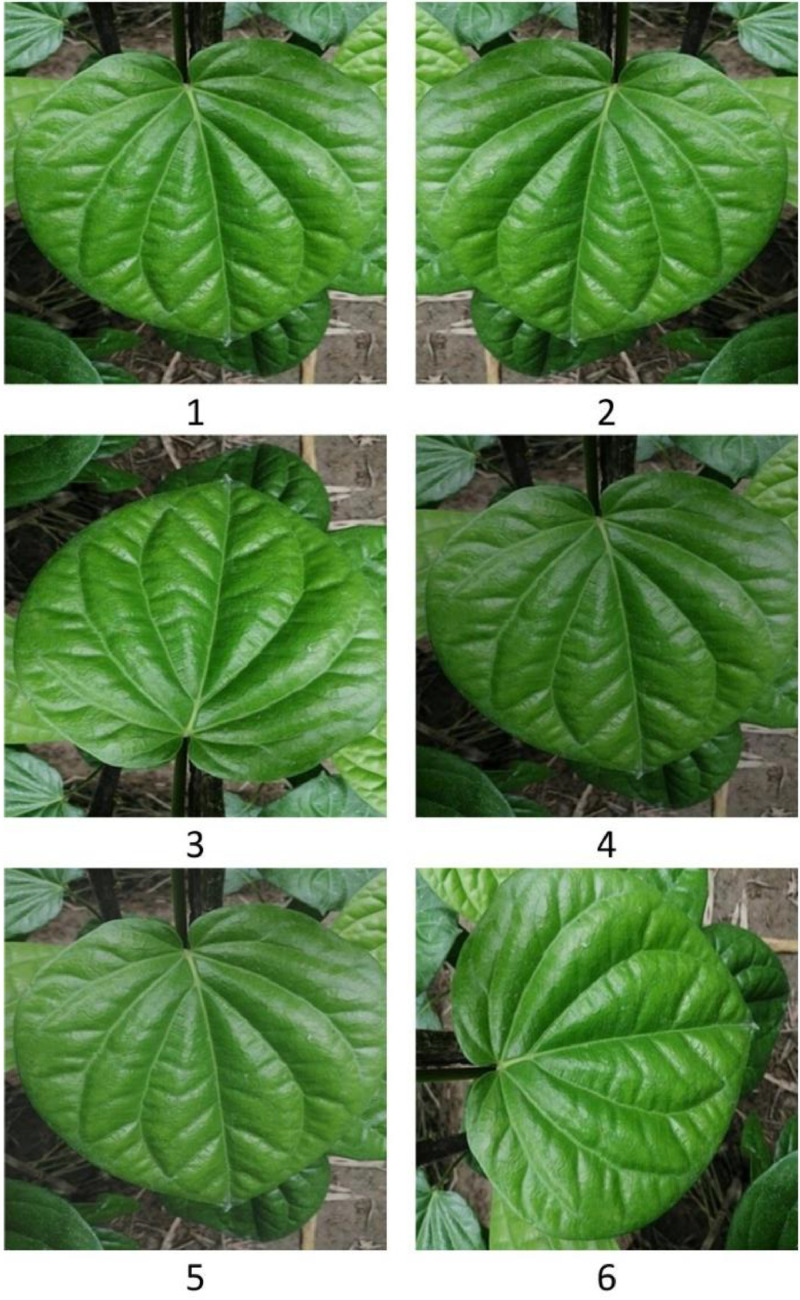
Augmented images of the betel leaf dataset. 1) original image, 2) horizontal flip, 3) vertical flip, 4) brightness factor, 5) contrast factor, and 6) rotation angle.

Afterward, images were reshaped to make images suitable for computer vision applications, and filenames were also renamed. After this, the original dataset was generated. Then image augmentation techniques were applied to collected images, these techniques were frequently utilized in related studies in the literature to generate relevant new images with diversity [15,16]. Brightness adjustment imitates several lighting conditions, contrast adjustment replicates natural variations in contrast, and flipping improves the invariance of models by helping in learning about orientation changes. Samples of generated images were also examined by pathology experts to ensure relevance and diversity. After the augmentation process, we got the augmented dataset of betel leaf. Finally, the process of the betel leaf dataset generation was completed. Details of augmentation techniques are given in Table 6 and in Fig. 4, representative images for each augmentation technique are shown along with the original image.

**Table 6:** Image augmentation strategies.

Strategies	Amount/Type	Description
Flipping [15,16]	Vertical, Horizontal	Flipping an image on a vertical axis or horizontal axis generates a mirrored version of the source image that improves the AI model capability of object recognition from different sides [17].
Brightness factor [15,16]	0.75	Changing the overall brightness of the real image creates a diversity of lighting conditions, that enhances the robustness and feature extraction capability of the AI model [17].
Contrast factor [16]	0.89	Changing the difference between the brightest and darkest parts of the source image to enhance or reduce the visibility of features, improves the efficiency of the model [17].
Rotation [15,16]	90 degrees	Rotating the orientation of the source image by tilting the image with a range of specified angles, improves AI models' generalizing capability to detect objects irrespective of their orientation [17].

**Fig. 5:**
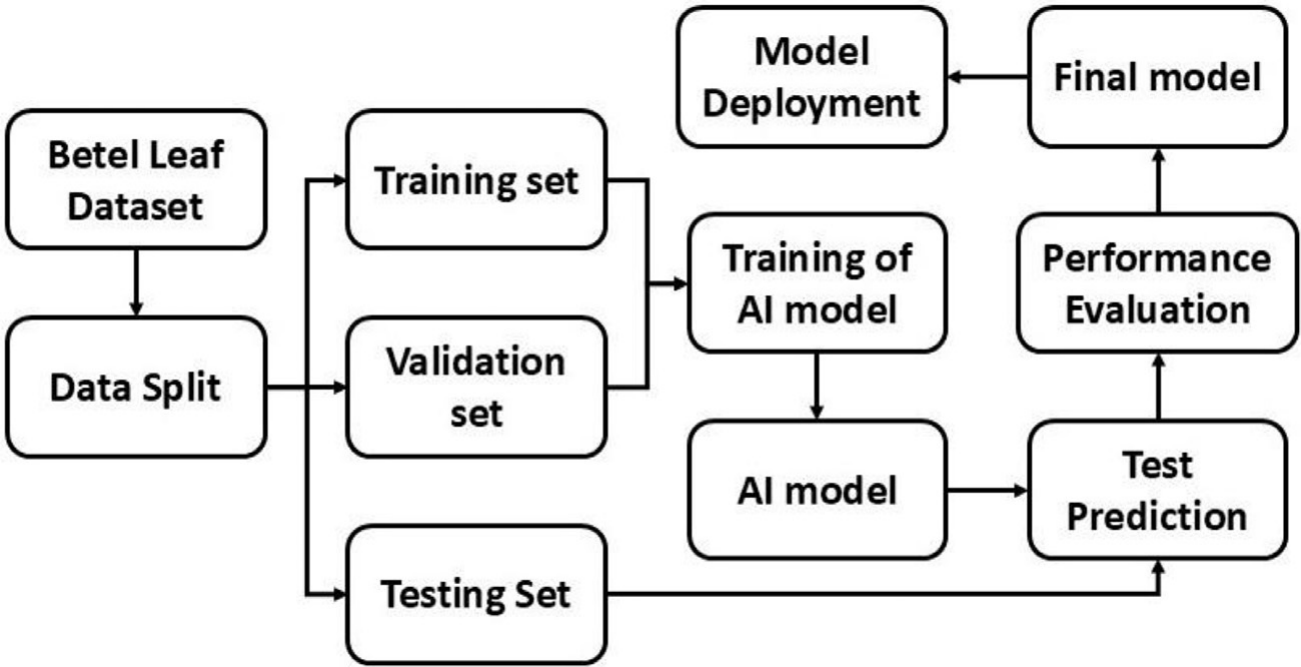
Workflow of the experimental design of betel leaf disease diagnosis.

Using the betel leaf dataset, several types of AI-based systems can be developed for betel disease diagnosis and management. A detailed workflow of the investigational setup for the betel disease diagnosis is shown in Fig. 5. By splitting sample images of the dataset into three different sets such as training, validation, and testing, researchers and interested individuals can build and evaluate AI models. The deployment of these models in IoT devices, smartphone applications, robotic systems, and expert systems can generate efficient tools for accurate disease diagnosis and management of betel leaf.

This dataset has several crucial implications for advanced AI-based research in the agriculture and plant pathology sector. The dataset can be utilized for developing efficient and accurate models for early disease diagnosis, that will assist farmers take preventive measures to enhance the quality of betel leaf. Accurate AI models generated with the presented dataset will also enhance production and crop management, which will reduce financial loss. This extensive dataset can be utilized to develop more robust and generalizable AI models, which can be deployed in mobile and IoT applications to support smart farming. This dataset can serve as a foundation for cross-domain adaptation and explainable AI in plant pathology research of betel leaf.

## Limitations

Images of this dataset were gathered from Mymensingh, Bangladesh that may not completely represent other varieties of betel leaf cultivates in other regions. Moreover, geographic diversity is low in Bangladesh, so this dataset may not work properly in other different geographic environments where soil, climate, and air conditions are different, even though the data collection process was conducted for two years and covers all climates of Bangladesh. This dataset contains leaf images of local betel plant varieties and only two common diseases found in Bangladesh that limit the area of research. Since leaf disease is highly common in Bangladesh, this dataset was merely focused on gathering images of betel leaves, and for this reason, it will not be appropriate for disease recognition of other parts of the betel plant. The leaf rot class has fewer images than other classes, which may bias overall model predictions, this can be solved using more data augmentation methods, class weighting, and resampling techniques.

## Ethics Statement

The authors thoroughly reviewed the ethical guidelines of Data in Brief and adhered to ethical prerequisites completely. We confirm that the whole dataset development process did not involve any experiments related to humans and animals and no data was gathered from any platforms of social media.

## CRediT Author Statement

**Rashidul Hasan Hridoy:** Conceptualization, Methodology, Investigation, Resources, Data Curation, Visualization, Writing - Original Draft, Writing - Review & Editing. **Md. Tarek Habib:** Conceptualization, Methodology, Supervision, Visualization, Writing - Review & Editing. **Imran Mahmud:** Validation, Writing - Review & Editing, Project administration*.* **Aminul Haque:** Formal Analysis, Writing - Review & Editing, Supervision. **Md Abdulla Al Mamun:** Writing - Review, Supervision.

## Data Availability

Mendeley DataComprehensive Betel Leaf Disease Dataset for Advanced Pathology Research (Original data).
